# Effect of Electropulsing Current Density on the Strength–Ductility Synergy of Extruded Mg-6Al-1Zn Alloy

**DOI:** 10.3390/ma18040751

**Published:** 2025-02-08

**Authors:** Dong Ma, Chunjie Xu, Yaohan Lu, Shang Sui, Jun Tian, Fanhong Zeng, Sergei Remennik, Dan Shechtman, Zhongming Zhang, Can Guo, Yuanshen Qi

**Affiliations:** 1 School of Materials Science and Engineering, Xi’an University of Technology, Xi’an 710048, China; 2 Xi’an Shechtman Nobel Prize New Materials Institute, Xi’an 710048, China; 3 Xi’an Key Laboratory of Advanced Magnesium Alloy Additive Manufacturing and Precision Forming, Xi’an 710048, China; 4 Guangdong Provincial Key Laboratory of Materials and Technologies for Energy Conversion, Department of Materials Science and Engineering, Guangdong Technion-Israel Institute of Technology, Shantou 515063, China; 5 MGM Nobel Prize New Materials Co., Ltd., Tongchuan 727100, China; 6 Center for Nanoscience & Nanotechnology, Hebrew University of Jerusalem, Edmond J. Safra Campus, Jerusalem 91904, Israel; 7 Department of Materials Science and Engineering, Technion-Israel Institute of Technology, Haifa 32000, Israel

**Keywords:** Mg-6Al-1Zn alloy, electropulsing treatment, microstructural evolution, mechanical properties

## Abstract

The difficulty in enhancing both tensile strength and ductility is limiting the development of high-performance Mg alloys. The “plastic deformation + electropulsing (EP) treatment” is an effective process for modifying the microstructure and enhancing the mechanical properties of metals. In this work, the influence of the current density of EP treatment on the microstructure and tensile property evolution of the as-extruded Mg-6Al-1Zn alloy was systematically investigated. The microstructure of the as-extruded sample was predominantly composed of an α-Mg matrix and a minor quantity of the β-Mg_17_Al_12_ phase on grain boundaries. After EP treatments, the microstructure underwent recrystallization, resulting in the formation of fine recrystallized grains. Meanwhile, the distribution and volume fraction of the β-Mg_17_Al_12_ phase demonstrated minor changes. After the 60 cycles of EP with a current density of 1050 A·mm^−2^ for a duration of 60 s, the tensile strength and elongation of the as-extruded Mg-6Al-1Zn alloy improved from 260 ± 2.6 MPa and 22 ± 1.3% to 319 ± 3.6 MPa and 23 ± 1.1%, respectively. The results prove the effectiveness of EP treatment in tailoring recrystallization via changing current density.

## 1. Introduction

Magnesium (Mg) alloys have been used extensively in a variety of fields, including transportation, aerospace, electronic communications, and the production of biodegradable materials, which are due to the lightest metallic structure and excellent mechanical properties [1,2]. However, the poor formability of the Mg alloy due to its hexagonal closed-packed crystal (HCP) structure and strong basal texture is a difficult problem that must be overcome in industrial applications [3]. In order to extend the applications of the Mg alloy, it is appropriate to modify the microstructure and improve the mechanical properties by thermomechanical processes and other treatments [4,5]. For instance, Li et al. [6] studied the thermal deformation of Mg alloy via dual-phase synergistically strengthening. The extruded alloy exhibited remarkable mechanical properties, including a yield strength of 372 MPa and an elongation of 12%. Ni et al. [7] investigated the as-extruded Mg-Al-Zn-Y sheets that exhibit high ductility, and the ultimate tensile strength was 321.3 MPa. It has been demonstrated that plastic deformation can enhance the mechanical properties of the material through the refinement of grain size and simultaneously reducing the defects within the microstructure in comparison to the cast ingots [8,9,10]. Nevertheless, the deformed Mg alloy components still exhibit considerable residual stress and a strong texture, thus requiring prolonged heat treatments to enhance their mechanical properties.

The electropulsing (EP) treatment has the advantages of high efficiency, low energy consumption, and remarkable effect, which is another effective material post-treatment method [11,12,13,14]. EP treatment can enhance the nucleation rate of recrystallization by applying pulse current to provide extra power and energy for recrystallization, thereby achieving the objective of grain refinement. In addition, the thermal and non-thermal effects generated by the electrical pulses can also facilitate dislocation movement and atomic and vacancy diffusion, as well as modify the microstructure of the alloy at relatively low temperatures [15,16,17]. Consequently, it is particularly suitable for deformed metal materials with high storage energy. Many studies have been conducted on the effects of EP treatment on mechanical properties [18,19,20], phase transformation [21,22,23,24], recrystallization [25], and the aging process [26,27]. Shan et al. [28] studied the Mg-Al-Zn alloy plates and achieved simultaneously improved strength and ductility with an ultimate tensile strength of 426 MPa and an elongation of 18.5% using EP treatment. Li et al. [29] demonstrated that combining EP treatment with subsequent aging treatment offers a new method to harness the advantages of both fine grains and a high density of nano-precipitates. This results in an increase in yield strength of approximately 50 MPa.

In summary, the combination of plastic deformation and EP treatment represents a promising approach for modifying the microstructure and enhancing mechanical properties. However, the mechanism of the EP process on the microstructure evolution and second phase precipitation behavior of magnesium alloys has not been clarified in previous studies. The customized EP treatments are still required to further optimize the microstructure to obtain better mechanical properties. This is particularly important given the potential formation of heterogeneous microstructures during the deformation process. The Mg-6Al-1Zn alloy is the most widely applied Mg alloy in industry, which has been extensively studied due to its excellent mechanical properties and machinability. Compared with other Mg alloys with complex compositions, the evolution of the primary phase Mg_17_Al_12_ in the Mg-6Al-1Zn alloy is more conducive to clarifying the influence of EP treatment. The as-extruded Mg-6Al-1Zn alloy was selected as the raw material and subjected to a series of different EP treatments in this study. The findings can serve as a valuable reference for the microstructure tailoring and the mechanical properties improvement of deformed Mg alloys.

## 2. Materials and Methods

Commercially pure Mg, Mg-30% Al, and Mg-30% Zn (purity > 99.9 wt. %) were used as the raw materials, and the Mg-6Al-1Zn (wt. %) alloy was fabricated in the SG2-3-10 vacuum melting furnace. The as-cast ingot was homogenized at 390 °C for 8 h and subsequently extruded at a rate of 0.5 mm·s^−1^ at 330 °C into strips with a width of 28 mm and a thickness of 1 mm (see Supplementary Figure S1). Finally, the strips were polished with 800^#^, 2000^#^, and 4000^#^ sandpaper before the EP treatments.

**Figure 1:**
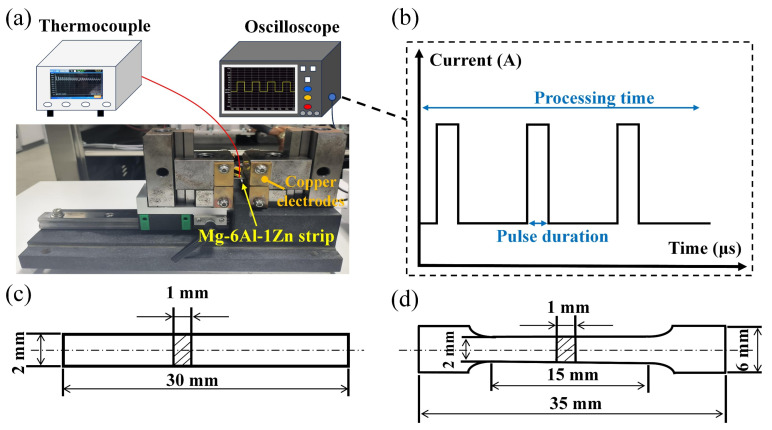
(**a**) The schematic view of EP system; (**b**) square-shaped wave form of EP treatment; (**c**,**d**) the geometries of the strip specimen and the tensile test specimen.

The EP processing system was composed of an EP generator, a digital storage oscilloscope, and a fixture that was composed of two Cu electrodes and a thermocouple, as illustrated in Figure 1a.

The EP process was performed by the EP generator, which could generate periodical rectangular current pulses (Figure 1b). The geometries of strip specimens and tensile test specimens were shown in Figure 1c,d. The dimensions of the tensile coupon were in accordance with ASTM E8M-09 and were classified as non-proportional specimens. During EP processing in the atmosphere, both kinds of specimens could be clamped by two Cu electrodes. In addition, the temperature at the center region of the sample was measured by a K-type thermocouple, as the Joule heat could dissipate via two Cu electrodes [14]. The EP parameters investigated were shown in Table 1.

In order to avoid adjusting too many parameters to clarify the mechanism of microstructure evolution, the current density parameter, which has the greatest influence weight, was selected. The main variable is the current density, namely, 705, 1050, and 1370 A/mm^2^ for EP-1, EP-2, and EP-3 samples, respectively. The number of EP cycles, single EP duration, and frequencies were set to be constant at 60, 1000 μs, and 1 Hz, respectively. Our study is dedicated to exploring the impact of electric current density on the evolution of the microstructure and tensile properties. For the microstructure characterization, the specimen surface parallel to the extrusion direction was polished using oxide polishing suspensions (OPS) solution. The second phase morphology of the central region of the samples was examined using the backscattering mode (BSD) of a scanning electron microscope (SEM) on a Zeiss Gemini 450 (Oberkochen, Germany). Electron backscatter diffraction (EBSD) measurements with a step size of 0.5 µm were conducted on an Oxford system. X-ray diffraction (XRD) with Cu Kα radiation was conducted in the 2θ range of 20–80° on a Rigaku MiniFlex (Tokyo, Japan). The tensile tests at room temperature were evaluated using the SANSI material test machine (Shenzhen, China) with a constant strain rate of 1 × 10^−3^ s^−1^.

## 3. Results

### 3.1. Mechanical Properties

**Figure 2:**
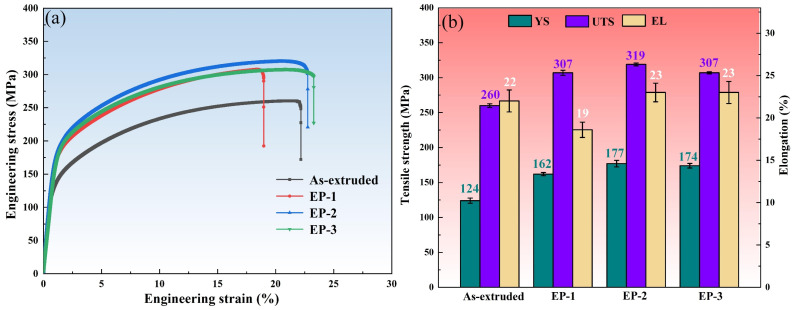
Representative mechanical properties of the as-extruded and EP-processed samples: (**a**) stress–strain curves; (**b**) tensile properties.

The mechanical properties of the as-extruded and EP-processed Mg-6Al-1Zn alloy at room temperature were evaluated by uniaxial tensile tests. Figure 2 shows the representative engineering tensile stress–strain curves and the corresponding ultimate tensile strength (UTS), yield strength (YS), and elongation (EL) of all the samples.

The as-extruded sample exhibited the UTS, YS, and EL of 260 ± 2.6 MPa, 124 ± 3.7 MPa, and 22 ± 1.3%, respectively, which was consistent with previous work [30,31]. After the EP-1 process treatment, the sample exhibited a slight decrease in ductility and a notable enhancement in strength, as the UTS and YS increased to 307 ± 3.6 MPa and 162 ± 2.3 MPa. With increasing current density, EP-2 and EP-3 samples exhibited further enhancement of both strength and ductility. The UTS, YS, and EL of EP-2 samples increased to 319 ± 2.0 MPa, 177 ± 4.7 MPa, and 23 ± 1.1%, respectively.

### 3.2. Microstructure Evolution

**Figure 3:**
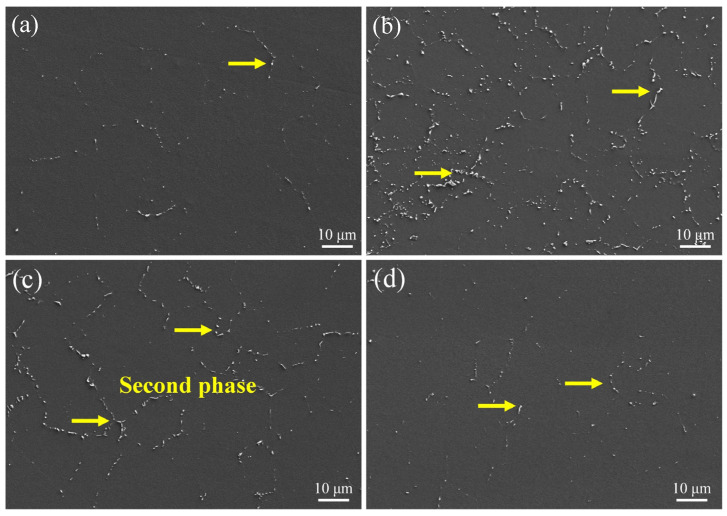
The microstructure evolution of the as-extruded Mg-6Al-1Zn alloy under varying EP treatments: (**a**) as-extruded; (**b**) EP-1; (**c**) EP-2; (**d**) EP-3.

Figure 3 shows the microstructure of the Mg-6Al-1Zn alloy samples with varying EP parameters. It is evident that the microstructure of the as-extruded sample primarily consisted of the α-Mg matrix and a minor quantity of bright white granular second phase distributed along the grain boundaries (yellow arrows, Figure 3a). The content of the second phase was 1.5 ± 0.2%. Following the EP-1 process, a substantial quantity of granular second phase precipitated within the microstructure (5.2 ± 0.7%, Figure 3b). As the current density increased, the content of the second phase decreased significantly to 3.4 ± 0.5%, as illustrated in Figure 3c. This may be attributed to the energy input by electropulsing, which resulted in the dissolution of the second phase. As the current density reached 1370 A·mm^−2^, the second phase content further decreased to 2.0 ± 0.4%.

**Figure 4:**
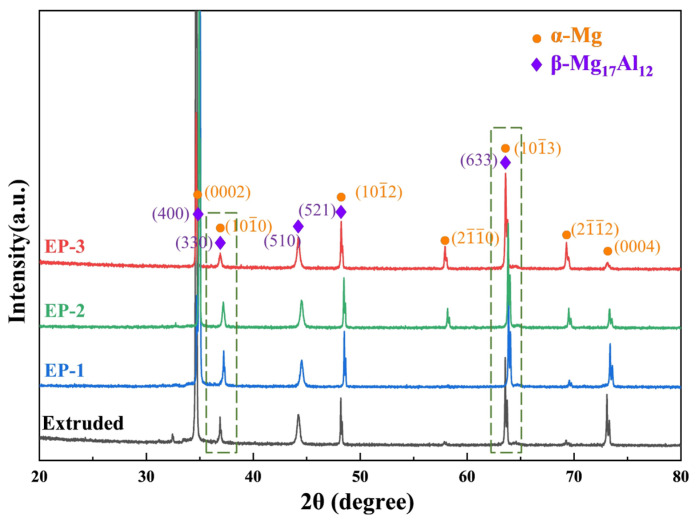
XRD patterns of the as-extruded Mg-6Al-1Zn alloy following different EP processes.

Figure 4 illustrates the XRD patterns of the as-extruded and EP-processed samples, demonstrating that the primary phases were α-Mg and β-Mg_17_Al_12_. After the EP treatments, the XRD peaks of α-Mg shifted correspondingly, which is due to the precipitation and dissolution of the solute elements. After EP-1 treatment, as more β-Mg_17_Al_12_ appeared, the content of Al as the solid solution decreased and led to a decrease in *d*-spacing and a shift of Mg peaks to higher angles. After the EP-2 and EP-3 treatments, with the gradual dissolution of the second phase, the Mg peaks shifted to lower angles. Eventually, for the EP-3 sample, the positions of the Mg peaks were nearly identical to that of the as-extruded one. This observation is consistent with the SEM characterization.

**Figure 5:**
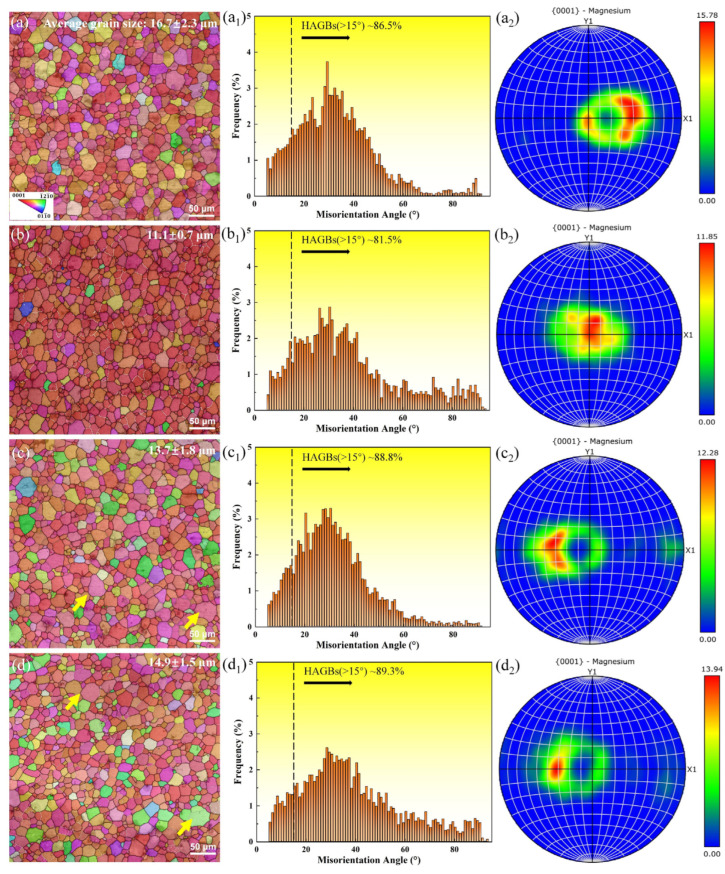
EBSD micrograph of the as-extruded and EP-processed samples: (**a**) as-extruded; (**b**) EP-1; (**c**) EP-2; (**d**) EP-3; (**a**–**d**) IPF images; (**a_1_**–**d_1_**) distribution of misorientation; (**a_2_**–**d_2_**) {0001} PF images.

Dense dislocations are usually generated within the material during the extrusion deformation process, providing the driving force for static recrystallization [32]. In order to study the effect of EP treatment, the as-extruded and EP-processed samples were characterized by EBSD measurements in Figure 5, which were perpendicular to the extrusion direction.

The microstructures of the as-extruded and EP-processed samples were composed of equiaxed grains of different sizes, namely the average grain sizes were 16.7 ± 2.3 μm for the as-extruded sample, 11.1 ± 0.7 μm after EP-1, 13.7 ± 1.8 μm after EP-2, and 14.9 ± 1.5 μm after EP-3, respectively (Figure 5a–d). In the case of the EP-1 sample, the storage energy present within the extruded material facilitated the occurrence of recrystallization, resulting in a reduction in grain size. The EP-2 and EP-3 samples exhibited greater energy due to the higher current density, which further promoted grain growth.

Figure 5a_1_–d_1_ presents statistical charts of the misorientation angle for all the samples. The proportion of high-angle grain boundaries (HAGBs) was 86.5% (as-extruded), 81.5% (EP-1), 88.8% (EP-2), and 89.3% (EP-3), respectively. In general, the HAGBs above 15° lead to the formation of easily pile-up dislocations, which indicates the ease with which plastic deformation occurs [33]. In addition, the as-extruded sample exhibited a basal texture {0001} with multiples of uniform distribution (MUD) values of 15.78, indicating a strong texture (Figure 5a_2_). Following the EP-1 treatment, it became evident that the crystallization phenomenon had occurred in the microstructure, resulting in a reduction in average grain size and a subsequent decline in MUD (11.85). Nevertheless, the EP-2 and EP-3 samples retained the basal texture, with MUDs (12.28 and 13.94) higher than that of the EP-1 sample. The average grain size in the EP-2 and EP-3 samples was larger, which had some coarse grains (yellow arrows, Figure 5c_1_,d_1_) and indicated slight grain growth occurring at relatively high current density.

## 4. Discussion

### 4.1. Effect of EP Treatment on Microstructure Evolution

#### 4.1.1. Effect of EP Treatment on Recrystallization

During the EP process, the temperature rise on the surface of the sample can be expressed by the following formula [34,35]:

(1) $$∆T=J^{2}\rho_{e}t/C_{P}\rho$$

where J is the current intensity of pulse current, $\rho_{e}$ is the resistivity of Mg, t is the electropuls duration, $C_{P}$ is the heat capacity, and ρ is the density of Mg. The actual temperatures of the EP-1, EP-2, and EP-3 samples measured by thermocouples were 80 °C, 115 °C, and 151 °C, respectively (Table 1). It is worth noting that due to the heat dissipation during the real experiment, the ∆T value can only be the maximum temperature rise in the experiment. This indicates that the experimental value was lower than the theoretical value. However, it can be seen that the temperature rise changes significantly with the increase in current density.

In general, the temperature of static recrystallization of a typical magnesium alloy is over 250 °C [36]. The EP-1 sample with a temperature rise of only 80 °C showed recrystallized fine grains. Therefore, it can be inferred that the non-thermal effect plays an important role in recrystallization. The non-thermal effect ($J_{a}$) of the pulse current can be described by Equation (2) [17]:

(2) $$J_{a}=\frac{ND_{1}Z^{*}e\rho J}{kT}$$

where N is the density of atoms, $D_{1}$ is the diffusion coefficient, $Z^{*}$ is effective valence of the solute Mg, e is the charge on an electron, k is the Boltzmann constant, and T is the absolute temperature. It can be inferred that the non-thermal effect caused by electropulsing is proportional to current intensity J, which significantly affects the microstructure. Unfortunately, the influence of complex physical mechanisms in non-thermal effects cannot be accurately quantified. Following the EP-1 treatment, recrystallization occurred in the microstructure, and the average grain size decreased, while the proportion of low-angle grain boundaries increased (Figure 5a,b). Consequently, the accumulation of high-density current and high dislocation density results in a greater non-thermal effect, which can facilitate the completion of static recrystallization in the EP-2 and EP-3 samples.

#### 4.1.2. Precipitation and Dissolution of β-Mg_17_Al_12_ Phase

EP treatment also has an effect on the evolution of the secondary phase. For the as-extruded sample, the β-Mg_17_Al_12_ phase was predominantly distributed along the grain boundaries, with a content of 1.5 ± 0.2%. After EP-1 treatment, a significant quantity of β-Mg_17_Al_12_ phase precipitated, with a content of 5.2 ± 0.7%. Meanwhile, the β-Mg_17_Al_12_ phase exhibited a clear spheroidization, as shown in Figure 3b. The transformation of the second phase is essentially a diffusion-controlled process, and the EP treatment increases the diffusion flux and electromigration ability of atoms and vacancies in the alloy [37]. In the low current density of the EP-1 treatment, the pulse current promotes the diffusion of Al atoms, resulting in the precipitation and dispersion of the β-Mg_17_Al_12_ phase. As demonstrated by Equation (2), the EP process significantly enhances the average atomic flux, which represents an additional high-energy input. The conventional solid solution heat treatment of the Mg-Al-Zn strip typically requires at least 30 min to complete within the temperature range of 673 to 693 K. However, the application of EP treatment results in the delivery of high-energy inputs to the crystal lattice of magnesium alloy in an instantaneous manner. At a relatively low temperature (323~343 K), the EP process results in the precipitation of the β-Mg_17_Al_12_ phase within tens of seconds, effectively inhibiting the growth of grains. Nevertheless, with the further increase in current density (EP-2 and EP-3 treatments), the second phase gradually dissolved into the matrix. The primary mechanism responsible for this phenomenon can be elucidated as follows: The high-density current significantly enhances the diffusion rate of metal atoms. The high-speed moving pulse electron flow produces a significant high-frequency periodic impact on the atoms in the metal, thereby raising the atoms to a relatively high-energy state. In this case, not only is the non-thermal effect augmented, but also the thermal Joule effect is increased. This results in an elevated sample temperature and an enhanced alloy intragranular diffusion coefficient.

**Figure 6:**
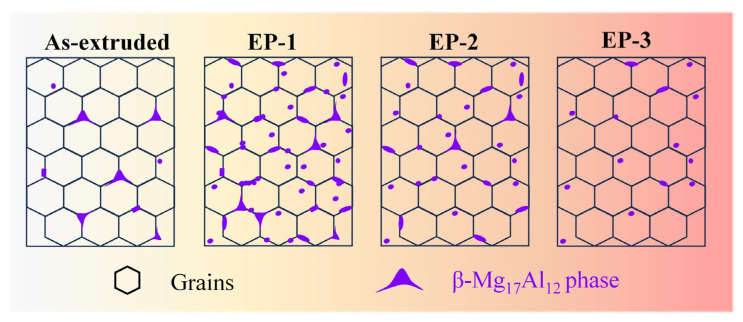
The evolution of that β-Mg_17_Al_12_ phase during the electric pulse treatment.

Furthermore, the thermodynamic driving force arises from the reduction in surface free energy at the interface. The Gibbs–Thomson effect posits that the concentration differential of Al in the α-Mg matrix, contingent upon the curvature radius corresponding to the β-Mg_17_Al_12_ phase, is as follows [38]:

(3) $$C_{r1}-C_{r2}=\frac{2V_{m}C_{0}\sigma }{RT}\cdot (\frac{1}{r_{1}}-\frac{1}{r_{2}})$$

where $C_{r1}$ and $C_{r2}$ are the concentrations of alloy element, respectively, corresponding to the curvature $r_{1}$ and $r_{2}$ of β-Mg_17_Al_12_ phase; $V_{m}$ is the molar volume; *σ* is the interfacial energy; *R* is the gas constant; and *T* is the absolute temperature. Equation (3) indicates that the concentration of alloying element in the vicinity of the β-Mg_17_Al_12_ phase is related to its radius. It reveals that as the radius of the second phase is reduced, the concentration of alloying element increases. The concentration difference between the Al elements in the β-Mg_17_Al_12_ phase and the surrounding material results in their diffusion from areas of low curvature (such as the tips and defects of the layers) to areas of high curvature (such as the plane of the layers). Consequently, the lamellar β-Mg_17_Al_12_ phase underwent dissolution and subsequent spheroidization during the EP process. Thus, the combined influence of the aforementioned factors resulted in the gradual dissolution of the β-Mg_17_Al_12_ phase. The evolution of that β-Mg_17_Al_12_ phase during the electric pulse treatment was illustrated in Figure 6. In conclusion, it can be demonstrated that EP treatment offers significant energy and time savings when compared with conventional heat treatment.

### 4.2. Strength–Ductility Enhancement Mechanism

Following the application of the EP treatments with different current densities the generation of fine recrystallized grains in the as-extruded alloy resulted in improved strength and ductility. The EP-2 sample exhibited the highest mechanical properties, and the UTS, YS, and EL were 319 ± 2.0 MPa, 177 ± 4.7 MPa, and 23 ± 1.1%, respectively. In addition, the recrystallized grains also contributed to reducing the anisotropy of the alloys so that the deformation can be more evenly dispersed in more grains, reducing the cracking tendency of Mg alloys and thus showing higher ductility.

However, compared with other EP-processed samples, the elongation of the EP-1 sample is notably inferior, even lower than that of the as-extruded sample. On the one hand, the recrystallization occurred after the EP-1 treatment, which contained more grains with low-angle grain boundaries (LAGBs). The LAGBs limited the deformation ability of the material. On the other hand, the interface between the β-Mg_17_Al_12_ phase and the α-Mg matrix is non-coherent, which results in a weaker precipitation strengthening effect and also makes it prone to becoming a crack initiation site. Furthermore, the precipitation of the second phase resulted in a significant reduction in the solid solution strengthening effect of the sample. Therefore, the elongation of the EP-1 specimen was poor, and the increase in tensile strength was limited. In comparison to the EP-1 sample, the recrystallization grains in EP-2 and EP-3, which have higher current densities, are mainly high-angle grains. Additionally, the second phase also underwent further dissolution. The solid solution strengthening effect was significantly enhanced, resulting in enhanced mechanical properties.

### 4.3. Customized EP Process Exploration and Application in Engineering

EP treatment has been shown to significantly change the microstructure of metal materials, such as recrystallization, reduction in dislocation density, and dissolution of the second phase, and suitable EP parameters can fully exploit the potential of the mechanical properties. Moreover, EP treatment can significantly enhance the mechanical properties and thermal stability of Mg alloys under high-temperature conditions, making them more suitable for use in extreme environments. By refining the grain structure and controlling the second phase, EP treatments provided important technological support for the application of Mg alloys in fields such as aerospace and automotive manufacturing. In this work, a customized EP treatment process exploration was proposed to achieve in tailoring recrystallization via changing current density. Compared to traditional treatment (for example, heat treatment), the EP process is simple, and the processing time is extremely short, usually only a few seconds or minutes, to achieve the same effect as several hours of heat treatment. Moreover, the EP-processed samples are not limited by size and shape, which only need to connect electrodes at both ends of the sample. Therefore, the application of EP can be widely used in engineering production. Based on its significant improvement in material performance, it can effectively enhance production efficiency and save energy. However, due to the characteristics of the pulse current, the temperature gradient of the sample during the EP process is not uniform, resulting in the EP treatment effect on the sample not being completely uniform. It may also cause a gradient difference in the microstructure. Therefore, the engineering application of EP treatment requires more extensive and in-depth research.

## 5. Conclusions

The effect of EP treatment on the microstructure evolution and enhancement of the mechanical properties of the as-extruded Mg-6Al-1Zn alloy strip was studied, and the following conclusions can be drawn:

1. The microstructure of the as-extruded sample primarily consisted of the α-Mg matrix and the β-Mg_17_Al_12_ phase. The content of the β-Mg_17_Al_12_ phase underwent changes in precipitation, followed by dissolution as the current density increased. The EP process accelerated the recrystallization behavior of the as-extruded sample.
2. Following the application of the EP treatments, the fine recrystallized grains and a controllable amount of β-Mg_17_Al_12_ phase were generated in the as-extruded alloy, which led to improved tensile properties. The EP-2 sample exhibited the highest mechanical properties, with the UTS, YS, and EL being 319 ± 2.0 MPa, 177 ± 4.7 MPa, and 23 ± 1.1%, respectively.
3. The rapid spheroidization and dissolution of the β-Mg_17_Al_12_ phase in the as-extruded sample, which is enhanced by the combined effect of thermal and athermal effects, are facilitated by the application of electrical pulses.

## Figures and Tables

**Table 1:** Studied samples and the electropulsing treatment parameters.

Sample	EP Cycles	Current Density (A·mm^−2^)	Pulse Duration (μs)	Frequency (Hz)	Measured Temperature (°C)
As-extruded	-	-	-	-	-
EP-1	60	705	1000	1	80
EP-2	60	1050	1000	1	115
EP-3	60	1370	1000	1	151

## Data Availability

The original contributions presented in the study are included in the article, further inquiries can be directed to the corresponding authors.

## Supplementary Materials

The following supporting information can be downloaded at: https://www.mdpi.com/article/10.3390/ma18040751/s1, Figure S1. The forward extrusion diagram of the Mg-6Al-1Zn strip.
