# Supplementary material for: Effect of Electropulsing Current Density on the Strength–Ductility Synergy of Extruded Mg-6Al-1Zn Alloy
Source: Materials (Basel). 2025 Feb 8;18(4):751. doi: 10.3390/ma18040751 (PMC11857845; doi:10.3390/ma18040751)
Supplement: Supplementary file 1 [file materials-18-00751-s001.zip › materials-3449666-supplementary.pdf]

Effect of Electropulsing Current Density on the Strength–Ductility Synergy of Extruded Mg-6Al-1Zn Alloy

The supplementary material contains one supplementary figure.

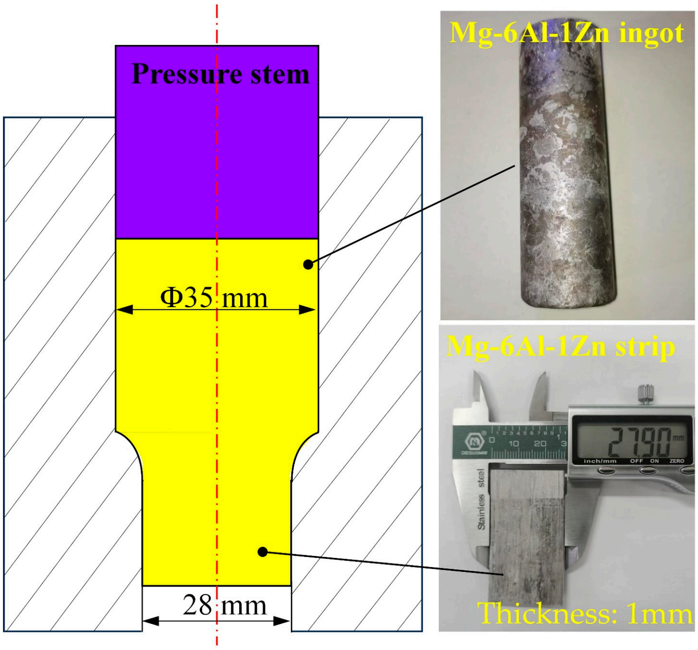

Figure S1. The forward extrusion diagram of the Mg-6Al-1Zn strip.
